# Living in endemic area for infectious diseases is associated to differences in immunosenescence and inflammatory signatures

**DOI:** 10.3389/fimmu.2025.1547854

**Published:** 2025-03-17

**Authors:** Monique Macedo Coelho, Felipe Caixeta Moreira, Luciana Werneck Zuccherato, Lucas Haniel de Araújo Ventura, Giovanna Caliman Camatta, Bernardo Starling-Soares, Lícia Torres, Danielle Fernandes Durso, Hugo Itaru Sato, Murilo Soares da Costa, Henrique Cerqueira Guimarães, Rafael Calvão Barbuto, Mauro Lúcio O. Júnior, Elaine Speziali, Unaí Tupinambas, Santuza Maria Ribeiro Teixeira, Gabriela Silveira-Nunes, Andrea Teixeira-Carvalho, Tatiani Uceli Maioli, Ana Maria Caetano Faria

**Affiliations:** ^1^ Departamento de Bioquímica e Imunologia, Instituto de Ciências Biológicas, Universidade Federal de Minas Gerais, Belo Horizonte, Brazil; ^2^ Departamento de Clínica Médica, Faculdade de Medicina, Universidade Federal de Minas Gerais, Belo Horizonte, Brazil; ^3^ Hospital Risoleta Tolentino Neves, Belo Horizonte, Brazil; ^4^ Hospital da UNIMED, Governador Valadares, Brazil; ^5^ Instituto de Pesquisa René Rachou, Fundação Oswaldo Cruz, Belo Horizonte, Brazil; ^6^ Universidade Edson Antônio Velano, Fundação de Ensino e Tecnologia de Alfenas, Belo Horizonte, Brazil; ^7^ Departamento de Medicina, Universidade Federal de Juiz de Fora, Governador Valadares, Brazil; ^8^ Departamento de Nutrição, Escola de Enfermagem, Universidade Federal de Minas Gerais, Belo Horizonte, Brazil

**Keywords:** endemic area, infectious diseases, immunosenescence, epigenetic clocks, environment

## Abstract

Research on aged individuals from developed countries show that lifestyle factors such as diet, physical activity, stress, smoking, and sleep quality impact aging. However, other relevant factors may influence aging in less-studied populations, such as Brazilian cohorts. This study aimed to analyze immunosenescence profile of individuals living in an endemic area for several infectious diseases in Brazil. We showed that these individuals exhibited accelerated epigenetic aging and increased production of IL-12p70, IL-17A, and IL-9. Production of inflammatory mediators IL-12p70, IL-6, IL-1β, IL-2, and IL-1ra in individuals with flu-like symptoms and those with COVID-19 was higher among residents in endemic areas than in residents from a control non-endemic area. Furthermore, residents of the endemic area had a more prominent inflammatory profile during viral infection and a different pattern of plasma mediators when compared to residents of a non-endemic area. Our data suggests that these two cohorts had specific immune signatures regardless of the presence or the type of infection at study. Therefore, we demonstrated that there were distinct patterns of immune responses and epigenetic aging depending on the environment the individuals live in. These observations add a layer of diversity to the studies of human aging by including individuals from less represented regions.

## Introduction

Aging is a multifactorial and heterogeneous biological phenomenon that affects individuals in diverse ways (1). Lifestyle factors, such as nutritional habits, physical activity, well-being, stress, smoking, and sleep quality, significantly influence the aging process (2, 3).

Aging affects all body compartments, and the immune system is one of the most affected. Immunological changes occurring during aging, which are collectively called immunosenescence, are associated with increased susceptibility to infections due to the progressive decline in immune function (4, 5). This process involves thymic involution with decreased production of naïve T cells, increased frequency of memory T cells, reduction in T cell repertoire and the accumulation of senescent and exhausted lymphocytes in the periphery (6). Immunosenescence also includes a chronic state of low-grade inflammation, termed “inflammaging,” characterized by alterations in the levels of plasma mediators such as IL-6, TNF-α, IL-1, and IL-10 (7, 8). Inflammaging is a systemic consequence of various aging-related events, including epigenetic changes, mitochondrial dysfunction, genomic instability, and the emergence of the senescence-associated secretory phenotype (SASP) by senescent cells (6).

Senescent T lymphocytes are cells that have undergone permanent cell cycle arrest, accumulate molecular damage, exhibit dysregulated metabolism, and produce senescence-associated mediators, in addition to expressing markers such as CD57 and TIGIT. These cells also lose essential co-stimulatory molecules, such as CD28 and CD27 (9). On the other hand, exhausted T lymphocytes are characterized by the expression of inhibitory co-receptors, such as PD-1, LAG-3, and TIM-3. They exhibit low proliferative capacity and reduced cytokine production (10). Furthermore, there is a decrease in the production of naive B lymphocytes, accompanied by the accumulation of oligoclonally expanded cells (11).

Despite their dysfunctional phenotype, Grosse and colleagues demonstrated that senescent cells play important physiological roles, and their elimination could lead to health deterioration and a shorter lifespan in mice due to the disruption of blood-tissue barriers (12). Moreover, not all aged individuals suffer from chronic inflammatory diseases and frailty. Studies on healthy nonagenarians and centenarians in Italy and in healthy elderly people in Brazil showed that these individuals develop remodeling mechanisms that control the deleterious effects of inflammation and immunosenescence (13–16).

Therefore, aging does not lead to a linear and universal decline in immune function. Immune remodeling mechanisms developed by some individuals allow them to adapt to the deleterious effects of immunosenescence (7). Studies conducted by our group showed that elderly individuals residing in endemic areas for schistosomiasis, but not infected (negative), develop innate immune protection mechanisms that compensate for the age-associated decline in T cell function (17, 18). One of these studies showed that the frequency of IFN-γ-producing NK cells increases with aging and remains elevated in individuals over 70 years old (17). In another study, we observed an increase in the frequency of NK cells expressing Toll-like receptors TLR-1,- 2, -3, and -4, as well as dendritic cells expressing TLR-1 and monocytes expressing TLR-1 and -4 (18). These findings suggest that compensation of the innate immune response may play a key role in resistance to infection and healthy aging in individuals residing in endemic areas.

However, we also reported that the enhanced innate responses triggered by continuous antigenic stimuli in these individuals from endemic areas may lead to accelerated senescence measured by methylation of CpG islands in the DNA (19).

DNA methylation is one of several mechanisms that regulate gene expression and is considered an important biomarker of aging. Based on methylation status, epigenetic clocks have been developed to calculate individuals’ biological age. These clocks can detect not only the acceleration of aging but also the effects of deceleration in models of healthy aging (20). Moreover, DNA methylation patterns are associated with a wide range of age-related diseases, including Alzheimer’s (21), cardiovascular diseases (22), and cancer (23). Age acceleration measured by methylation patterns are known to be altered in response to environmental stimuli such as exercise (24), diet (25), smoking (26), and pollutants (27). One study demonstrated that an eight-week treatment program, including diet, sleep, exercise, relaxation techniques, and probiotic supplementation, was able to reduce the epigenetic age of a group of 43 adult men by 3.23 years (28).

Herein, we examined aging in an endemic area (EA) in Brazil, where the prevalence of schistosomiasis, leishmaniasis, leprosy and viral infections is high. This study was conducted based on two points of departure. First, immunosenescence has been known as a consequence of chronic antigenic exposure (29), but there is no detailed evaluation of the immunological repercussions in the immunosenescence profile of individuals residing in EAs compared to those living in non-endemic areas (NEAs). Second, as mentioned before, previous reports by our group showed that healthy aging in such endemic areas are achieved at the cost of augmented innate immune responses that provide resistance to infectious agents (17, 18).

Traditionally aging studies focuses on cohorts and issues associated with developed countries and Caucasian populations. Our hypothesis is that, in addition to lifestyle, other significant environmental factors such as chronic exposure to infectious agents may be associated with distinct patterns of immunosenescence and aging. Therefore, in this study, we aimed to evaluate the immunological repercussions in a cohort of individuals residing in an endemic area for infectious diseases in Brazil.

## Materials and methods

### Ethics statement

This study meticulously adhered to the ethical standards set forth by National Research Ethics Committee (CONEP), prioritizing the safety and well-being of all participants. It was approved by Human Research Ethical Board of UFMG (CEP-UFMG) and the National Research Ethics Committee (CONEP) (CAAE # 40208320.3.1001.5149). All volunteers were informed of the objectives and procedures involved in the study and they signed the Informed Consent Form (TCLE) before their voluntary participation.

### Study subjects

The research was conducted in two distinct Brazilian cities: one living in a non-endemic area for infectious diseases (Belo Horizonte/MG), where 107 participants were enlisted, and another in an endemic region (Governador Valadares/MG), also with 107 participants. This amassed a cohort of 214 individuals, whose attributes are shown in **Table 1**.

**Table 1:** Characterization of the study sample.

	AE (endemic area)			NEA (non-endemic area)		
	Flu- Like n = 45	COVID-19 n = 47	Negative Control n = 15	Flu- Like n = 45	COVID-19 n = 47	Negative Control n = 15
Age (years)	38 (18-60)	38,5 (18-77)	41 (19-78)	36 (19-63)	40,5 (18-76)	39 (18-65)
Sex Female (%)	51,1	46,8	46,7	46,6	53,19	53,3
Sex Male (%)	48,9	54,2	53,3	53,4	46,80	46,6
BMI (Kg/m²)	28,3 ± 5,57	26,29 ± 4,53	26 ± 4,21	28,4 ± 7	30 ± 5,67	27 ± 6,82
Viral Load (ct)	_	21,89 ± 7,12	_	_	27,3 ± 5,14	_
Comorbidity (%)	28,8	23,4	20	24,4	23,4	26,6
Hipertension (n)	9	6	2	6	6	3
Diabetes Miellitus (n)	3	3	0	4	3	1
Respiratory Disease (n)	0	2	1	1	0	0
Others (n)	1	0	0	0	2	0

NEA- non-endemic area; EA- endemic area; NC- negative control; FS- flu syndrome; BMI – body mass index calculated by the formula kg/m^2^ where kg is a person’s weight in kilograms and m^2^ is their height in squared meters.

Volunteer recruitment spanned from December 2020 to October 2021, focusing on identifying individuals displaying symptoms akin to flu-like syndromes of unknown origin (Flu-like syndrome) and COVID-19, alongside including individuals in good health (control group). Throughout the collection period, circulating strains of COVID-19 encompassed both the original variant and P1. All chosen volunteers underwent an RT-PCR (Real-Time Polymerase Chain Reaction) test to validate the diagnosis of COVID-19. Volunteers were recruited at UPA-Centro Sul and Hospital Universitário Risoleta Tolentino Neves in Belo Horizonte and Hospital Unimed in Governador Valadares.

The study’s inclusion criteria involved recruiting adult or elderly volunteers aged 20 years old or above, irrespective of whether they exhibited flu-like symptoms, provided they consented to sign the Informed Consent Form (TCLE). The participant selection process also adhered to specific exclusion criteria, excluding children and adolescents (under 18 years old), individuals with inconclusive results in the RT-PCR test for COVID-19 detection, flu-like symptoms persisting beyond 9 days, and individuals living with HIV.

The body mass index (BMI) of the participants was categorized based on the following criteria: less than 18.5 (underweight), between 18.5 and 24.9 (normal weight), between 25.0 and 29.9 (overweight), and exceeding 30.0 (obesity).

The volunteers were engaged by the study team in health units where they reviewed and addressed any queries regarding the Informed Consent Form (ICF), they underwent a clinical and sociodemographic questionnaire session and had their blood samples collected for serum and plasma separation, alongside swab collection for SARS-CoV-2 detection via RT-PCR. Individuals diagnosed with COVID-19 were telemonitored for 14 days following confirmation and delivery of the RT-PCR test results.

### Luminex-multiplex measurement of inflammatory mediators

To assess blood mediators, we utilized heparinized plasma from our volunteers, employing the Bio-Plex^®^ Pro Human Cytokine Standard multiplex kit by Bio-Rad Laboratories. This kit facilitates the simultaneous analysis of numerous analytes through magnetic immunoassay, conducted on Luminex equipment (Bio-Plex^®^ 200, Bio-Rad). The quantified panel of analytes encompasses IL-1β, IL-1Ra, IL-2, IL-4, IL-5, IL-6, IL-7, CXCL8, IL-9, IL-10, IL-12p70, IL-13, IL-15, IL-17A, CCL11, FGF-Basic, G-CSF, GM-CSF, IFN-γ, CXCL10, CCL2, CCL3, CCL4, PDGF-BB, CCL5, TNF-α, and VEGF. Analyses was conducted utilizing the Bioplex™ xPONENT software version 3.1 by Bio-Rad.

### Radar plots

The radar chart depicted cytokines/chemokines categorized as low (≤ global median) and high (≥ global median) producers, utilizing the global median of each mediator as a cut-off (30). The calculation of the global median encompassed the entire dataset derived from the respective groups. Within the radar, each axis symbolizes individuals exhibiting elevated levels of a specific mediator (termed high producers). These axes interconnect to form a polygonal area, illustrating the equilibrium between growth factors, inflammatory, and anti-inflammatory mediators. An increase or decrease in this area signifies a higher or lower contribution of each mediator to the overall profile.

### DNA extraction and bisulphite treatment

Genomic DNA extraction was conducted from peripheral whole blood samples using the Trizol and Chloroform protocol. Subsequently, 1 μg of DNA underwent bisulfite conversion utilizing the EZ-96 DNA Methylation Kit (Zymo Research, Irvine, USA) with specific adjustments: incubation in CT buffer for 21 cycles of 15 minutes at 55°C and 30 seconds at 95°C, followed by elution of the bisulfite-treated DNA in 100 μl of water. Post-extraction and conversion, the samples were loaded onto the Illumina Infinium Methylation EPIC Bead Chip. Fluorescence data were collected from all enrolled subjects, and subsequent beta values were calculated.

### Estimation of DNAm age

Following the acquisition of the methylation beta value matrices file, we utilized the methylclock 1.5.0 package (31) to estimate biological ages and aging acceleration through nine distinct methylation clocks.

### Serology

Serological analysis was carried out on individuals from EA (Governador Valadares, Brazil) and from NEA (Belo Horizonte, Brazil) to detect IgG antibodies against the dengue virus and cytomegalovirus (CMV). All examinations were outsourced to the Hermes Pardini Laboratory in Belo Horizonte, Brazil, adhering to the institution’s protocols for detection techniques, sample transportation, and storage methods. Dengue serology employed Enzyme Immunoassay, facilitating the quantitative detection of specific IgG for all dengue serotypes (DEN1, 2, 3, and 4). CMV testing utilized the ELFA (Fluorimetry Enzyme Assay) technique, allowing the quantitative detection of IgG antibodies specific to CMV types 1 and 2 antigens.

### Spearman’s correlation analysis

Spearman’s coefficient analysis was performed using the R package to assess multiple associations between biomarkers and determine the strength of relationships. The correlation coefficient value ranges from -1 to +1, where a positive value indicates changes in the same direction and a negative value indicates opposite directions. A value of 0 signifies no association between variables. The correlation plot displays only those correlations with p < 0.05. Circle size represents statistical significance, with larger circles denoting lower p-values and increased significance.

### Statistical analysis

The significance of differences between groups was assessed through parametric tests (Student’s t-test and analysis of variance – ANOVA, followed by Tukey’s post-test) or non-parametric tests (Mann-Whitney U and Kruskal-Wallis test, followed by Dunn’s post-test). The normality of sample distribution was assessed using the Kolmogorov-Smirnov test. Participant characteristics were presented as mean (standard deviation) or median (minimum to maximum range) for continuous variables and as frequency (number) for categorical variables. When appropriate, means (standard deviation) or medians (interquartile range) were depicted in graphs. All statistical analyses were conducted using the GraphPad Prism 8.0 package, considering a difference as significant when p < 0.05.

## Results

### Individuals residing in endemic areas had accelerated epigenetic aging

**Figure 1:**
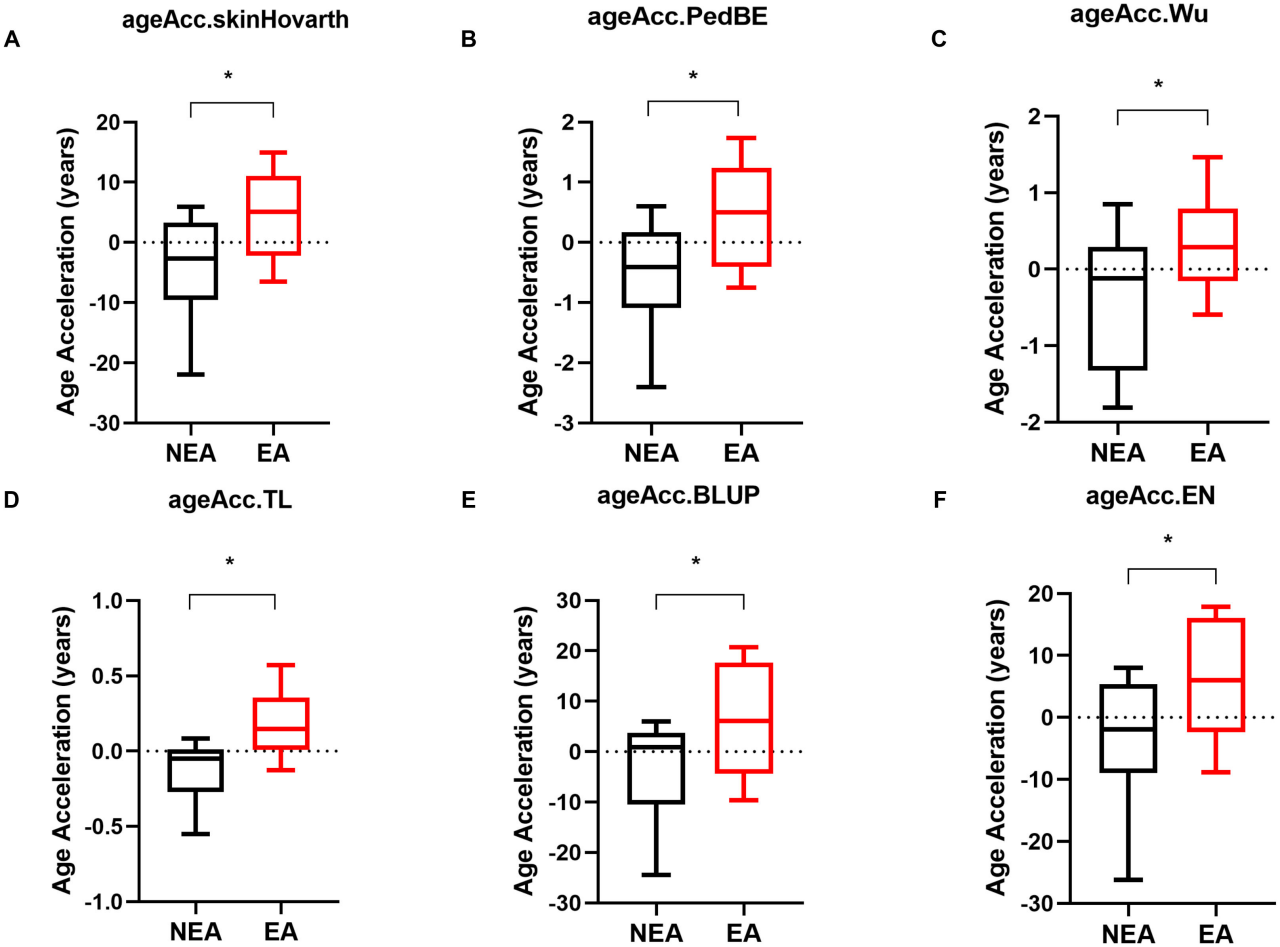
Individuals residing in EA exhibit accelerated epigenetic aging compared to those in NEA. **(A-F)** Acceleration of biological aging in individuals residing in NEA represented by black boxplots (n=11) compared to individuals residing in EA represented by red boxplots (n=11). The skinHovarth, PedBE, Wu, TL, BLUP, and EN clocks from the methylclock 1.5.0 package of the R software were utilized. The unpaired t-test was conducted. *p ≥ 0.05.

A previous report conducted by our group described that individuals living in an area endemic for infectious diseases had accelerated biological age measured by DNA methylation (19). This study included volunteers from Governador Valadares, MG, an endemic area (EA), and individuals from São Paulo, SP, a non-endemic area (NEA). In this study, we also compared the epigenetic aging of the volunteers from another city, Belo Horizonte, MG, as a NEA sample. Epigenetic analyses indicated that individuals living in EA also exhibited accelerated aging compared to those living in NEAs across the six biological clocks evaluated: skinHovarth (**Figure 1A**), PedBE (**Figure 1B**), Wu (**Figure 1C**), TL (**Figure 1D**), BLUP (**Figure 1E**), and EN (**Figure 1F**).

### Individuals residing in endemic areas for infectious diseases presented higher production of IL-12p70, IL-17A, and IL-9

**Figure 2:**
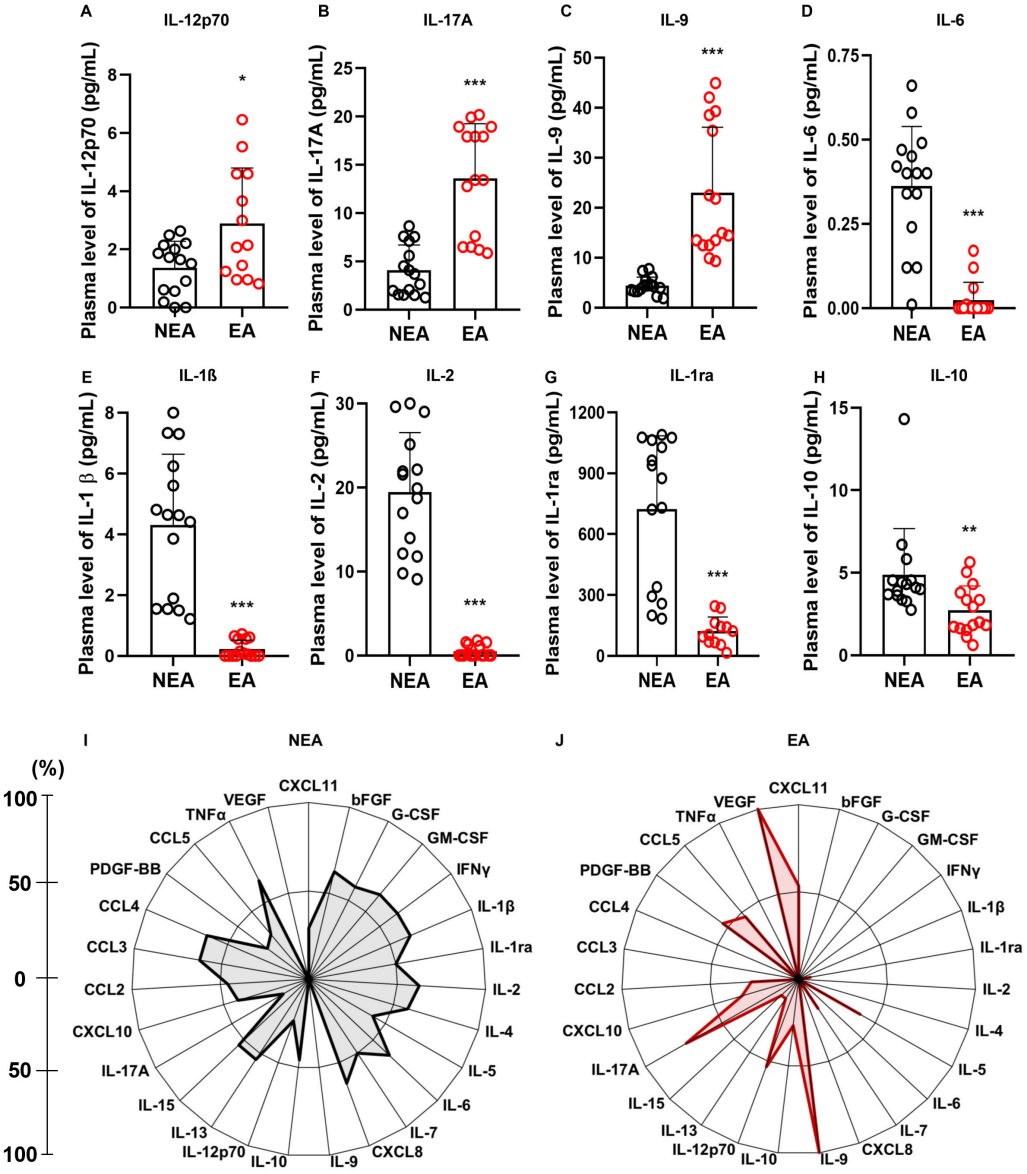
Individuals residing in EA exhibit differences in inflammatory mediators: **(A)** Comparison of IL-12p70 production, **(B)** IL-17A, **(C)** IL-9, **(D)** IL-6, **(E)** IL-1β, **(F)** IL-2, **(G)** IL-1ra, and **(H)** IL-10 production between individuals residing in NEA (black bars, n=15) and those residing in EA (red bars, n=15). **(I)** Radar plot illustrating the production of inflammatory mediators in individuals residing in NEA (n=15). **(J)** Radar plot representing the production of inflammatory mediators in individuals residing in EA (n=15). In the radar plots, each axis represents the percentage (%) of volunteers exhibiting a high frequency of inflammatory mediator production, as indicated by the line on the left. The central polygonal area reflects the overall profile of each group. Inflammatory mediator production was assessed using the Luminex assay. Statistical differences were determined using the Mann-Whitney test, with significance denoted as * = p ≤ 0.05, ** = p ≤ 0.01, *** = p ≤ 0.001.

Since aging can lead to various alterations in the immune response (6), we investigated the immune repercussions in the production of inflammatory mediators in our cohort. Residents in EA had higher production of IL-12p70 (**Figure 2A**), IL-17A (**Figure 2B**), and IL-9 (**Figure 2C**) compared to individuals living in NEA. On the other hand, residents in NEA showed higher production of IL-6 (**Figure 2D**), IL-1β (**Figure 2E**), IL-2 (**Figure 2F**), IL-1ra (**Figure 2G**), and IL-10 (**Figure 2H**). Additionally, individuals residing in EA presented a less homogeneous profile and a higher frequency of high producers of PDGF-BB, VEGF, CXCL11, IL-9, IL-12p70, and IL-17A compared to NEA residents (**Figure 2I, J**).

Notably, more than 60% of these individuals tested positive for CMV and 80% were reactive to Dengue virus as opposed to individuals from a non-endemic area where only 6% of individuals were positive for CMV and Dengue infections (**Table 2**).

**Table 2:** Prevalence of CMV and Dengue infections among negative control (NC) individuals residing in the NEA and EA based on serological analysis.

	Negative control individuals residing in NEA (n=15)	Negative control individuals residing in NEA (n=15)
CMV	6%	66,6%
Dengue	6%	80%

### Individuals from endemic area with either flu-like symptoms or COVID-had higher production of plasma mediators

**Figure 3:**
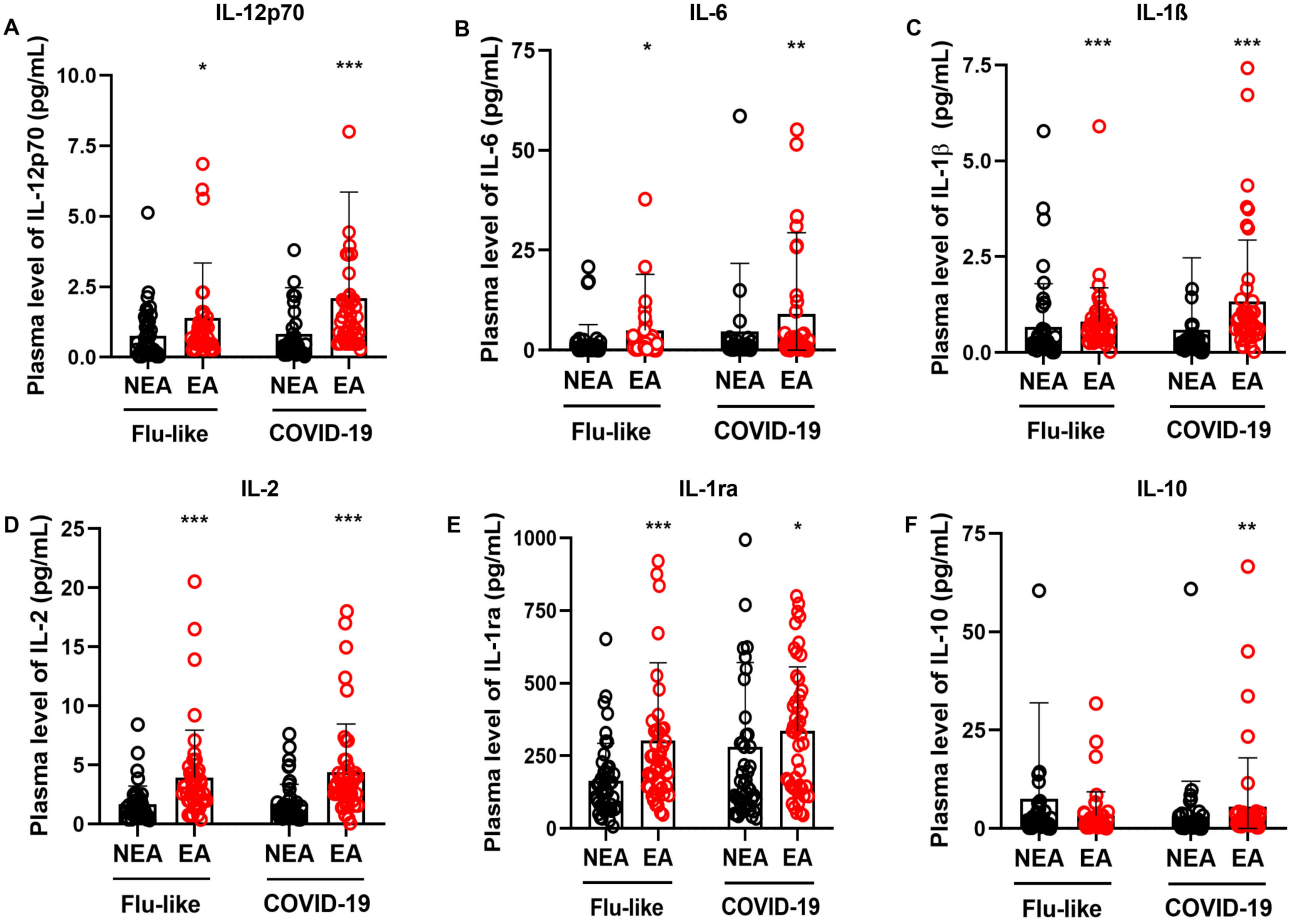
Individuals with flu-like symptoms or COVID-19 residing in EA exhibit higher production of inflammatory mediators. **(A)** Comparison of IL12-p70 production, **(B)** IL-6, **(C)** IL-1β, **(D)** IL-2, **(E)** IL-1ra, and **(F)** IL-10 in individuals with flu-like symptoms (n = 45) or COVID-19 (n = 47), residing in NEA, depicted by black bars, and individuals residing in EA, depicted by red bars. The production of inflammatory mediators was assessed using a Luminex assay. The Mann-Whitney test was conducted, and statistical differences were denoted by * = p ≤ 0.05, ** = p ≤ 0.01, *** = p ≤ 0.001.

Alterations in immune activity associated with aging can increase vulnerability to infectious diseases (32). In this study, we explored putative differences in the production of inflammatory mediators in response to viral infections between the two cohorts. Participants from endemic areas for infectious diseases (EA) were compared to those from non-endemic areas (NEA) who exhibited either flu-like symptoms or COVID-19. Individuals with either flu-like symptoms or infected with COVID-19 residing in EA exhibited higher production of IL-12p70 (**Figure 3A**), IL-6 (**Figure 3B**), IL-1β (**Figure 3C**), IL-2 (**Figure 3D**), and IL-1ra (**Figure 3E**) compared to NEA residents. Additionally, EA individuals with COVID-19 showed increased production of IL-10 compared to NEA individuals (**Figure 3F**).

### High producer profile of individuals residing in endemic areas versus non-endemic areas

**Figure 4:**
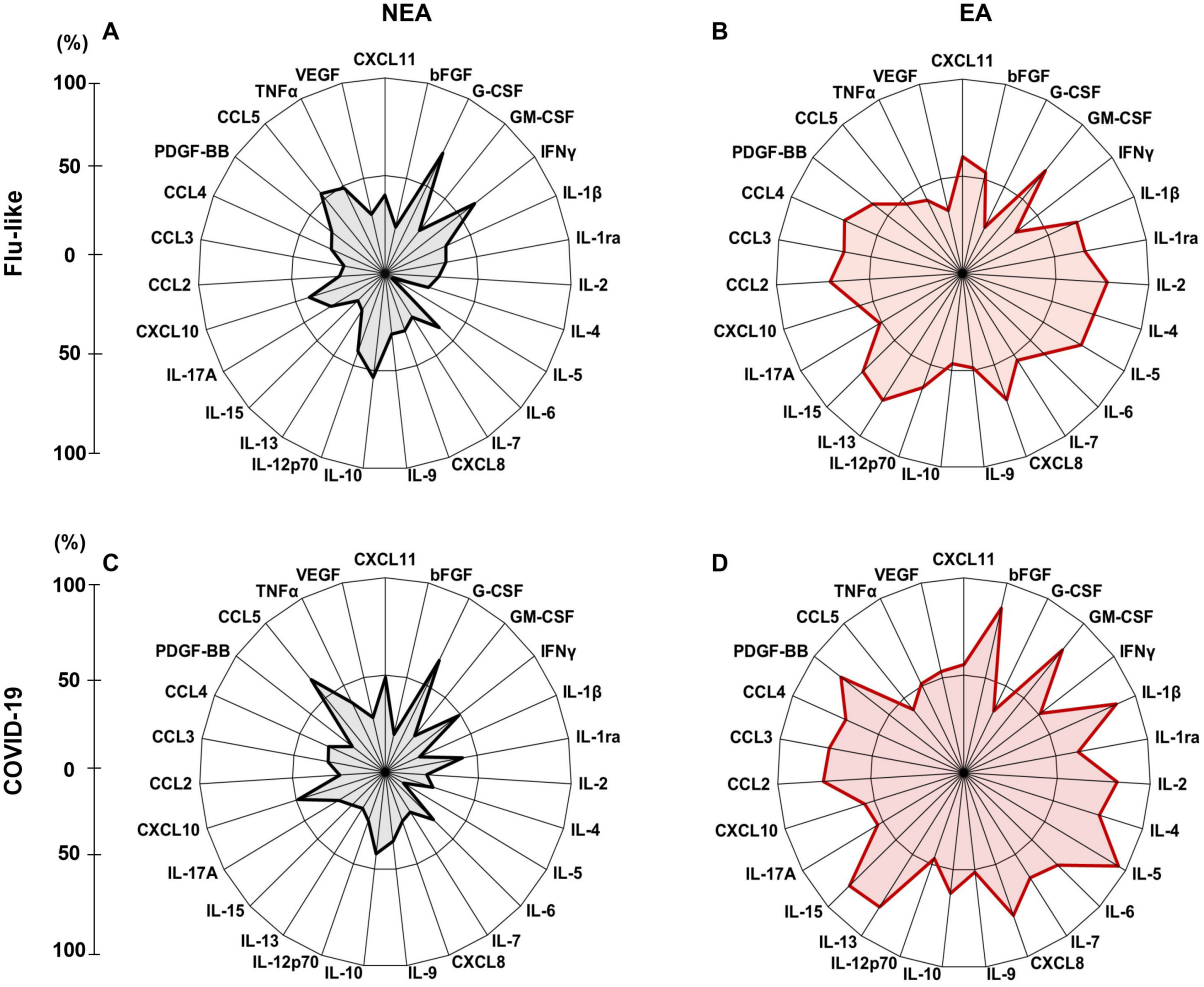
Infected individuals residing in EA exhibit a elevated frequency of high producers of inflammatory mediators: **(A)** Radar plot illustrating the production of inflammatory mediators in individuals with flu-like symptoms residing in NEA (n = 45). **(B)** Radar plot representing the production of inflammatory mediators in individuals with flu-like symptoms residing in EA (n = 45). **(C)** Radar plot showing inflammatory mediator production in individuals with COVID-19 residing in NEA (n = 47). **(D)** Radar plot displaying inflammatory mediator production in individuals with COVID-19 residing in EA (n = 47). In the radar plots, each axis represents the percentage (%) of volunteers exhibiting a high frequency of inflammatory mediator production, as indicated by the line on the left. The central polygonal area reflects the overall profile of each group. Inflammatory mediator production was assessed using the Luminex assay.

In addition to the differences found in cytokine production, we observed that these individuals exhibited a higher frequency of high producers of inflammatory mediators, anti-inflammatory mediators, chemokines, and growth factors compared to NEA residents, both with flu-like syndromes (**Figure 4A, B**) and COVID-19 (**Figure 4C, D**). Furthermore, the inflammatory profile of individuals residing in EA is very similar to the profile of individuals residing in NEA regardless of the type of infection, (**Figure 4C, D**).

### Individuals residing in endemic area had distinct correlations among inflammatory mediators and epigenetic age

**Figure 5:**
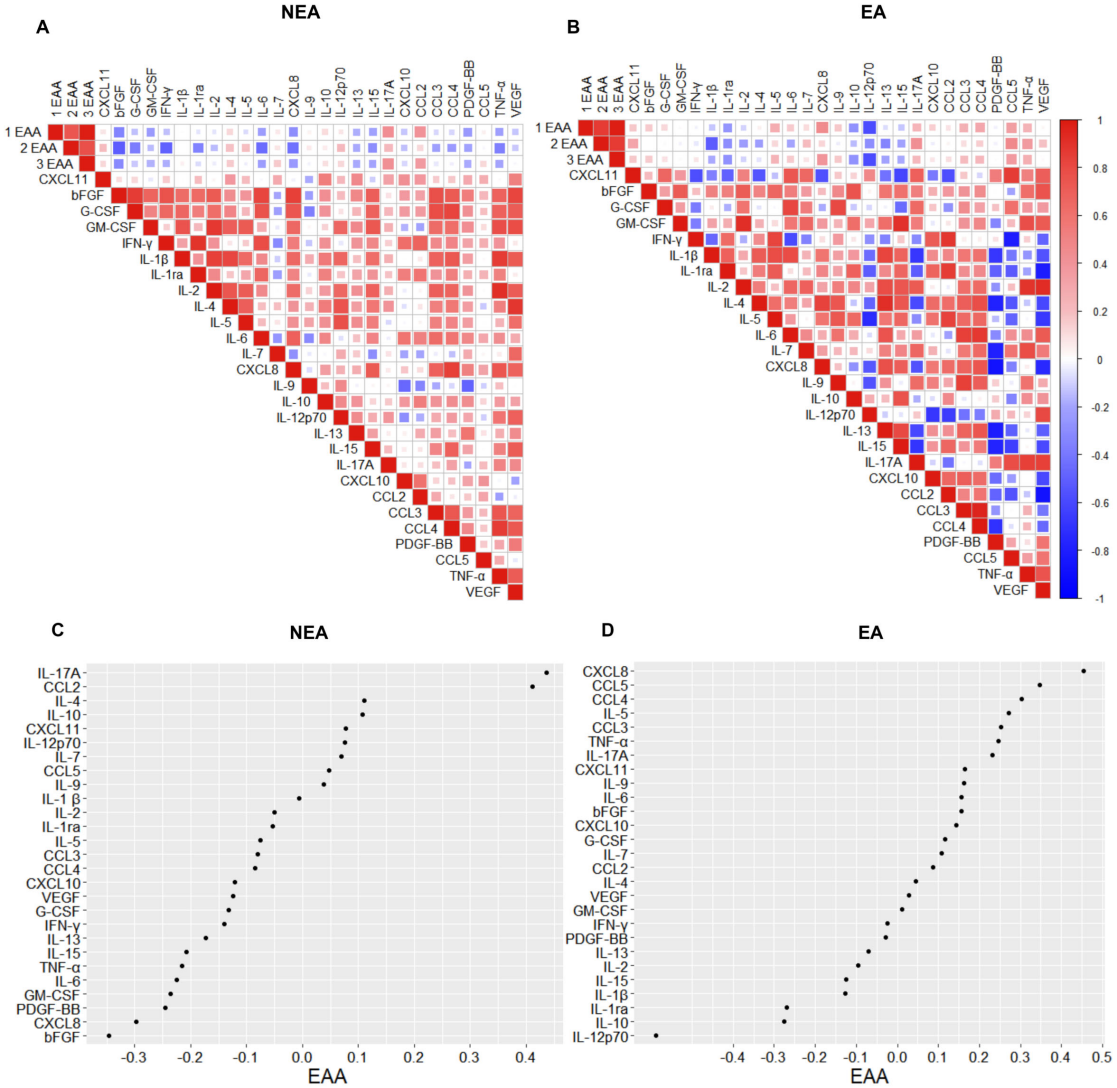
Individuals residing in EA exhibit distinct correlations of immunological phenotypes associated with epigenetic aging. **(A)** Spearman correlograms depicting plasma cytokines, chemokines, and growth factors correlation with the acceleration of biological aging (1EAA= skinHovarth, 2EAA= PedBE, and 3EAA= BLUP) in individuals residing in NEA and **(B)** EA (n = 11). The intensity of the correlation between two variables is represented by the color of the square at their intersection, ranging from bright red (strong positive correlation; i.e., r2 = 1.0) to bright blue (strong negative correlation; i.e., r2 = -1.0). **(C)** Spearman correlogram between the acceleration of biological aging (EAA= skinHovarth) and the production of cytokines, chemokines, and plasma growth factors in individuals residing in NEA and **(D)** EA (n = 11). The display shows NEA correlations from 0.4 to -0.3 and EA from 0.5 to -0.5. Data represent p-values ≤ 0.05 for statistical analysis conducted between groups.

Aging is accompanied by a state of low-grade chronic inflammation, known as “inflammaging” (6, 7, 33). Since individuals residing in EA exhibit accelerated aging and possess a distinct profile in the production of inflammatory mediators compared to NEA residents, we aimed to understand the biomarkers associated with the biological aging of these individuals by comparing correlation matrices. The correlogram of individuals living in EA showed more intense positive correlations among inflammatory mediators than the correlogram of individuals living in NEA (**Figure 5A, B**). Furthermore, epigenetic age is positively correlated, in increasing order, with CXCL8, CCL5, CCL4, IL-5, CCL3, TNFα, IL-17A, CXCL11, IL-9, IL-6, bFGF, CXCL10, G-CSF, IL-7, CCL2, IL-4, VEGF, and GM-CSF in EA volunteers, while in NEA volunteers, epigenetic age is positively correlated, in increasing order, with IL17A, CCL2, IL-4, IL-10, CXCL11, IL-12p70, IL-7, CCL5, and IL-9 (**Figure 5D**).

## Discussion

Environmental factors, such as lifestyle, can play a crucial role in promoting healthy aging. While chronological aging progresses at a fixed rate, the rate of biological aging varies among individuals. Biological age can be measured by epigenetic clocks, which are based on DNA methylation patterns (20).

To understand whether frequent exposure to infectious antigens leads to accelerated aging, we used six epigenetic clocks to compare the aging of individuals residing in an EA with those in a NEA. Our results showed that individuals in the EA exhibit accelerated epigenetic aging across all evaluated methylation clocks (**Figure 1A-F**). These findings are consistent with results from a previously published study by our group, despite using a cohort from a different NEA (19).

Among the various modifications associated with aging and immunosenescence, one of the most prominent is the “inflammaging” phenomenon characterized by the increased systemic production of IL-1, IL-6, and TNF-α (6). In this study, differently from the previous one, we examined the production of inflammatory mediators in two cohorts of individuals and found that residents in EA had increased production of IL-12p70 (**Figure 2A**), IL-17A (**Figure 2B**), and IL-9 (**Figure 2C**). In contrast, individuals residing in the NEA showed higher production of IL-6 (**Figure 2D**), IL-1β (**Figure 2E**), IL-1ra (**Figure 2F**), IL-2 (**Figure 2G**), and IL-10 (**Figure 2H**).

Although our results indicate higher production of IL-6 (**Figure 2D**) and IL-1β (**Figure 2E**) in NEA residents—cytokines typically associated with *inflammaging* - it is important to note that the volunteers in both groups were age-matched. Furthermore, this study does not focus on a cohort of elderly individuals but rather on two cohorts with distinct biological aging profiles. Moreover, aging is a continuous process that occurs throughout life, beginning at birth.

IL-12 consists of the p35 and p40 subunits, which combine to form the bioactive IL-12p70. This cytokine is crucial for IFN-γ production and the activation of Th1 cells (34). IL-17A, on the other hand, is primarily produced by Th17 cells in response to IL-1β and IL-23, mediating immunity against fungi and bacteria (35). IL-9 is a pleiotropic cytokine produced by Th2 cells, ILC2s, mast cells, and basophils, playing a significant role in type 2 immunity, autoimmunity, and the immune response to tumors (36). IL-6 is an acute-phase pro-inflammatory cytokine secreted primarily by macrophages, important for regulating inflammatory disorders such as viral infections (37, 38). IL-1β, also produced by macrophages, is cleaved by inflammasome activation, which is triggered by caspase-1. Besides protecting against pathogens, this cytokine regulates sterile insults and plays a crucial role in proliferation, differentiation, and apoptosis (39). Conversely, IL-1ra acts as a natural inhibitor of IL-β by serving as an antagonist of the interleukin-1 receptor (40). Increased production of IL-1ra is probably a regulatory response to control the elevated secretion of IL-1β. IL-10 also exerts potent immunosuppressive functions (41). On the other hand, IL-2 can have opposing functions playing a significant role in both inflammation and suppression. It is involved in the proliferation of T cells, facilitating the generation of effector and memory cells, and in the maintenance of regulatory T cells (42).

In response to immunogenic challenges, the immune response typically polarizes towards Th1, Th2, or Th17 pathways. Interestingly, our data revealed a lack of polarization in the profile of mediators from individuals residing in EA with elevated levels of inflammatory cytokines associated with diverse immune functions. In contrast, the panel of mediators produced by individuals in NEA was more balanced, with elevated production of both early inflammatory cytokines and anti-inflammatory cytokines. This suggests that at steady state individuals residing in endemic areas had an immunological inflammatory profile very distinct from the one exhibited by individuals from non-endemic areas.

Inflammation is a natural process that plays a crucial role in various functions, including embryogenesis and tissue repair (43). A balanced profile of immune response is considered a marker of health since chronic inflammation is detrimental and can contribute to biological aging, as observed in studies of individuals infected with CMV (44), autoimmune diseases such as systemic lupus erythematosus (45), and conditions like obesity or type 2 diabetes (46). Despite the differences observed in the production of inflammatory mediators between the groups, their inflammatory status remained subclinical, and no symptom was reported. Moreover, participants in both groups do not exhibit significant comorbidities or differences in age or sex (**Table 1**). However, more than 60% of these individuals test positive for CMV infection and 80% for dengue infection in serological assays (**Table 2**). It is likely that these infections influence the inflammatory profile of EA individuals (47).

It is known that changes in immune activity associated with aging can increase vulnerability to diseases (9, 32, 48). Therefore, we also investigated whether individuals residing in endemic areas exhibited differences in the production of inflammatory mediators in response to viral infections. We compared residents of EA with those in NEA who had flu-like symptoms or were diagnosed with mild COVID-19 according to the World Health Organization (WHO, 2020) classification criteria.

Residents of EA with COVID-19 and FLS had higher production of IL-12p70, IL-6, IL-1β, IL-2, and IL-1ra compared to residents of NEA (**Figures 3A-E**), as well as an increased production of IL-10 in COVID-19 patients (**Figure 3F**). Additionally, these individuals showed a greater frequency of high producers of cytokines, chemokines, and growth factors, compared to those in NEA, both among those with flu-like symptoms (**Figures 2A, B**) and those with COVID-19 (**Figures 2C, D**).

Therefore, individuals residing in EA not only responded more intensely to infections than those in NEA, but they also produced a distinctive panel of mediators during these responses. We hypothesize that this may be due to a combination of an immunosenescent inflammatory profile, characteristic of those exposed to multiple infections throughout life (**Figure 2**), and their response to the viral antigens they encountered at the time of the study. A previous report supports this hypothesis, as it demonstrated that even young adults co-infected with cytomegalovirus and Epstein-Barr virus already had an aging-related T cell phenotype (49).

One of the most significant changes associated with aging is the emergence of cells with reduced activity and proliferative capacity, along with a senescence-associated secretory phenotype (SASP). These cells produce inflammatory cytokines and play a role in the inflammatory process (33, 50). Lifelong exposure to infectious antigens promotes chronic activation of the immune system, contributing to the emergence of senescent T cells, which are part of the SASP and accompany the aging process (51). Additionally, exhausted T cells also accumulate with age. These cells are characterized by the expression of inhibitory receptors and a reduced capacity to produce cytokines, along with impaired effector function after encountering an antigen (10). These cellular phenotypes are associated with compromised immune responses to vaccination, increased frailty, and greater susceptibility to age-related diseases (50).

Additionally, our results showed that although individuals with COVID-19 residing in EA produced higher levels of mediators compared to those with flu-like symptoms (**Figures 4C, D**), the inflammatory profile of EA residents was very similar regardless of the type of infection they carry (**Figures 4B, D**). The same was true for residents in NEA (**Figures 4A, C**).

These results lead us to believe that environmental differences, triggered by local stressors, were associated not only with varying rates of biological aging but also with distinct immunological profiles at steady state and during viral infectious diseases.

To understand whether there are differences in aging-associated inflammatory mediators between individuals from EA and those from NEA, we correlated three epigenetic clocks with the production of inflammatory mediators. The correlation matrix for individuals living in EA showed a greater number of positive correlations and stronger correlations among inflammatory mediators compared to the correlogram for individuals in NEA (**Figures 5A, B**).

Additionally, epigenetic age was positively correlated, in descending order, with CXCL8, CCL5, CCL4, IL-5, CCL3, TNF-α, IL-17A, CXCL11, IL-9, IL-6, bFGF, CXCL10, G-CSF, IL-7, CCL2, IL-4, VEGF, and GM-CSF among EA volunteers (**Figure 5D**). In contrast, for NEA volunteers, epigenetic age was positively correlated, in descending order, with IL-17A, CCL2, IL-4, IL-10, CXCL11, IL-12p70, IL-7, CCL5, and IL-9 (**Figure 5C**).

It has been proposed that aging is not characterized solely by deleterious changes and functional decline, but it includes a remodeling process to cope with challenges that occurs throughout life (6–8, 52). In this sense, aging involves compensatory changes resulting from the immunological history of individuals. Our data on the immunological and biological age of individuals who are residents of EA and NEA confirm this hypothesis.

Several studies indicate that aging can be accelerated by various lifestyle factors, such as processed foods and poor quality (2). In this study, we added a layer of complexity to these findings demonstrating that living in endemic areas for infectious disease may also impact on the immunological profile of individuals and in their aging process. Continuous exposure to strong antigenic stimulation is probably determinant for these differences, and they may be an important factor for the aging process of populations from many regions of the world, not only Brazil. Therefore, these data are relevant for a more universal understanding of immunosenescence and for the design of strategies to promote healthy aging.

The present study had limitations. Although the samples were matched for sex, age, and comorbidities, the sample size was small, particularly in the groups of individuals who did not exhibit flu-like symptoms and tested negative for COVID-19. Furthermore, immunophenotyping to assess senescent and exhausted cells would be crucial for a better understanding of the immunosenescent profile of these individuals. These factors highlight the need for future studies with larger sample sizes to confirm the findings.

## Data Availability

The original contributions presented in the study are included in the article/supplementary materials. Further inquiries can be directed to the corresponding author/s.
